# The coexistence of traditional medicine and biomedicine: A study with local health experts in two Brazilian regions

**DOI:** 10.1371/journal.pone.0174731

**Published:** 2017-04-17

**Authors:** Sofia Zank, Natalia Hanazaki

**Affiliations:** Laboratory of Human Ecology and Ethnobotany, Department of Ecology and Zoology, Federal University of Santa Catarina, Trindade, Florianópolis, Santa Catarina, Brazil; Missouri Botanical Garden, UNITED STATES

## Abstract

This study investigated the combined use of traditional medicine and biomedicine by local experts in Chapada do Araripe communities (Ceará State) and maroon communities (Santa Catarina State), Brazil. The objective was to understand the perception of local health specialists regarding the number of healers, demand for healers and use of medicinal plants, and the dependence of different environments to obtain such plants. We also aimed to understand the role of medicinal plants to treat different categories of diseases and if there is a complementary use of medicinal plants and allopathic biomedicine, according to the context of each group. The research was conducted with local health specialists that answered structured interviews, created free lists and participated in guided tours to collect cited plants. Sixty-six local health specialists were identified in the Araripe communities and 22 specialists in the maroon communities. In the maroon communities, a greater number of specialists thought there was a decrease in the number and demand for healers, as well as the use of medicinal plants, due to changes in traditional livelihoods, since they are located in a region where the effects of the modernization were more intense. In the Chapada do Araripe communities the specialists knew more plants extracted from native vegetation, whereas in the maroon communities cultivated plants were better known, which may reflect the environmental conditions and the history of each region. Medicinal plants are preferred to treat simpler health problems that do not require medical care, such as gastrointestinal problems, general pain, flues and colds. The biomedicine is used principally for problems with blood pressure, general pains and endocrine and nutritional diseases. Even with the particularities of each region, in general the use of medicinal plants and biomedicines occurred in a complementary form in both regions; however, this coexistence may result from these different contexts. This study also found that there was knowledge and appreciation for traditional health practices in both regions.

## Introduction

Traditional medicine is defined as “the sum total of the knowledge, skills, and practices based on the theories, beliefs, and experiences indigenous to different cultures, used in the maintenance of health as well as in the prevention, diagnosis, improvement or treatment of physical and mental illness” [1]. In Brazil, as well as many other Latin American countries, traditional medicine was historically built from a combination of knowledge and practices of different peoples, especially indigenous groups, Europeans and Africans [2, 3, 4]. The comprehension of a situation opposite to what is considered adequate health has been locally called many terms, such as disease, sickness, affections and health problems. The present work uses the term disease to designate different perceptions of changes in health.

The use of medicinal plants and the demand for local health specialists stand out among the diverse practices of traditional medicine that are found in both rural areas and urban centers [3, 5, 6, 7, 8]. Local health specialists are people of communities that are the most knowledgeable about traditional medicine and often about medicinal plants [2, 6, 8, 9, 10, 11], and, for this reason, they play an important role in the appreciation and dissemination of this knowledge. Healers, medicinal plant specialists and midwives are some examples of local health specialists [12].

Medicinal plants that were customarily cultivated or extracted from native vegetation are increasingly being purchased in local markets, pharmacies and other establishments. Purchasing these plants has gradually become more common in communities with easy access to urban centers and in areas where there are traditional markets that have historically commercialized these types of resources [13, 14, 15].

The effects of biomedicine, here defined as the hegemonic medical system based on the principles of Western science, on traditional medicine have been the focus of ethnobotanical studies over the past few decades [11, 16, 17, 18 19, 20]. Some authors, such as Ngokwey [21] and Saethre [22], consider biomedicine as one the main threats to traditional medicine. Access to biomedicine, mainly the use of allopathic medicines, may be the biggest reason for the reduction in the use and loss of knowledge of medicinal plants in indigenous communities [16, 21, 22]. However, in some situations traditional practices and biomedicine can coexist and complement each other [17, 19], and in remote and impoverished areas biomedicine is frequently part of a pluralistic medical system that includes self-care using medicinal plants and consulting local specialists [16]. We are considering as complementarity systems those that rely on both biomedicine and traditional medicine, depending on the type and severity of the diseases, and to social and cultural factors.

This study investigated the use of traditional medicine and biomedicine in communities from two distinct regions, the semiarid region of northeastern Brazil and the coastal region of southern Brazil. These regions have different socioenvironments and historical backgrounds, but in both areas there is access to biomedicine and it is possible to perceive changes in traditional health practices. Both areas also share the rural background that is gradually changing in the last few decades towards a more urbanized infrastructure. Based on the perception of local experts, who are guardians of knowledge of traditional medicine, we sought to evaluate the effect of biomedicine in traditional medicine through the following questions: 1) Are there changes in the use of traditional health practices (healers and use of medicinal plants)?; 2) Are there changes in the form of obtaining the medicinal plants, such as preference to purchase plants from pharmacies and local markets, instead of extraction or cultivation?; and 3) Does complementarity occurs in the use of herbal and allopathic medicines by local experts? Based on these questions, the perception of local specialists was analyzed in relation to changes in the number of healers, demand for healers and use of medicinal plants. Different disease categories treated by medicinal plants were investigated, as well as the use of different environments to obtain plants, and the most cited plants. Whether the use of allopathic medicines and medicinal plants occurred in a complementary manner and if traditional practices are threatened by biomedicine were also investigated, drawing attention to similar processes and especially to contextual differences (environmental, cultural, and historical) between both studied regions.

## Material and methods

### Study area

The study was conducted in two areas with communities that use traditional health practices but have access to biomedicine: rural communities in the Chapada do Araripe, in northeastern Brazil, and maroon (*quilombola*) communities in coastal southern Brazil. In the Chapada do Araripe communities there is a strong dependence on natural resources as a source of income, mainly from extracting resources from the natural environment but also from agriculture. The maroon communities are located near urban centers, but maintain traditional livelihoods within their territories. The main traditional health practices in the two regions are the use of medicinal plants and visiting healers that treat a diversity of diseases with the aid of prayers and plants. In both regions the access to biomedicine systems has been facilitated since the 1980s, both through the increase in the access to electricity and technology, and through government programs that improved the accessibility of remote areas and increased investments in health services for the population. Even with similar macroprocesses, both regions may have been affected differently due to historical, cultural, and environmental issues at each site. Ceará state, where Chapada do Araripe communities are located, has a medium Municipal Human Development Index (MHDI) of 0.682 (the 17th place among 27 Brazilian states) and 24% of the population living in rural areas, while Santa Catarina state, where maroon communities are located, presents a high MHDI of 0.774 (3rd place among 27 Brazilian states) place. human development index, and only 16% of the population living in rural áreas [23].

#### The Chapada do Araripe Communities

Chapada do Araripe is located on the border of the states of Ceará, Pernambuco and Piauí, is known for its environmental and cultural diversity, and has been the focus of various ethnobotanical studies [24, 25, 26, 27]. The region is in the Caatinga domain, with several types of vegetation, including areas of *cerrado*, *cerradão*, humid forest and *carrasco* [28]. Three communities were selected in the state of Ceará: Macaúba, Cacimbas and Maracujá, which depend on the natural environment to maintain their livelihoods [12] and are representative of the heterogeneity of the communities in the region. Macaúba is located on the slope of a hill, around 14 km from the center of the municipality of Barbalha, and has a population of circa 275 families. Cacimbas is located on the plateau of the Chapada, circa 15 km from the center of the municipality of Jardim, and has a population of approximately 260 families. Maracujá is located on the plateau of the Chapada, comprises three small adjacent communities (Baixa do Maracujá, Cruzeiro and Santo Antônio), is circa 20 km from the center of the municipality of Crato, and comprises circa 500 families. In Macaúba and Maracujá there is public transport to the center of the cities, but Cacimbas lacks this service.

In the past, communities of Araripe lived mainly from extraction of plant products and subsistence agriculture. Cassava, beans, corn and sugarcane were the major crops in the region. From the 1970s and 1980s, with the improvement of access roads to the center of cities and access to electricity, the process of modernization and modification of traditional livelihoods began, and some people started to look for paid employment. On the other hand, due to the remote location in the countryside of northeastern Brazil, the effects of modernization and government programs have still not impacted strongly upon traditional livelihoods. Campos *et al*. [29] verified that in these communities the dependence on extraction of plant resources is still string, and about 70% of the population of Cacimbas practice extractivism of plant resources, 60% in Maracujá and 40% in Macaúba.

In relation to health, in the late 1970s the vaccination campaigns and the improvement of roads begun, providing to the communities the access to biomedical resources (medical care, hospitals, and pharmacies). The first hospital in the region opened in 1936 in Crato, but it was accessible to people from the studied communities after the road improvements. In the early 1990s, the federal government launched a programme providing qualified community health workers to attend to primary health care, making home visits and providing guidance to local families. Moreover, in the 2000s a health center was built in each community, facilitating the access to biomedical resources.

#### The maroon communities of coastal Santa Catarina

The communities are located in the central-south coastal region of Santa Catarina State, in the municipalities of Garopaba (Morro do Fortunato and Aldeia communities) and Paulo Lopes (Santa Cruz community), and were recognized as maroon between 2006 and 2010 [30]. The region is in the Atlantic Forest domain and covered with vegetation that varies from dense forest to restinga. Presently, the maroon communities are trying to get their territory legally recognized and to have their traditional practices related to their African heritage better valued, including knowledge related to health.

The Morro do Fortunato community has approximately 30 houses and 90 residents, is located in a region on the side of a hill and is surrounded by native vegetation. Santa Cruz has circa 30 houses, 130 residents and is located over 1 km from a road in a rural neighborhood of Paulo Lopes. Aldeia is located in an urban region of the Garopaba municipality and has approximately 35 houses and 120 residents.

Previously, the communities lived mainly off small scale agriculture (cassava, maize, beans, peanuts, sugarcane, and sweet potatoes), fishing, and extraction of plant products. In the 1970s the main road connecting the south and northeastern coast of Brazil was constructed, creating easy access to the capitals of the southern states and a consequent expansion of tourism. Since then the livelihoods of the local communities has changed in a quick pace. Currently, in the three communities, a small percentage of adults have income associated with agriculture, animal husbandry, and/or fishing (16% in Fortunato, 2% in Santa Cruz, and 6% in Aldeia), and formal and informal urban jobs provide the majority of the income [31].

In relation to health care, the first hospital in the region exists since 1856, in Laguna (a municipality about 60 km far from the communities), but access to it was difficult. Since the 1970s and 1980s, the improvement of roads and the construction of health centers in the municipalities facilitated the access to biomedicine. In the late 1990s, the programme of the federal government provided health workers to attend to primary health care through home visits to local families.

### Methods

The study was authorized by the Research Ethics Committee at the Universidade Federal de Santa Catarina (authorizations 01128112.0.0000.0121 on 10/09/2012 —Chapada do Araripe—and 18847013.0.0000.0121 on 14/08/2013 —maroon communities) and participation of the informants was conditional on the signature of the written term of free informed consent. Access to traditional maroon knowledge was also authorized (process IPHAN 01450.012607/2013-20).

Data collection was made with local specialists from the two regions. We understand by local specialists, or local experts, are those who stand out in their knowledge of traditional medicine, mainly related to the practice of blessing and use of medicinal plants. The sampling strategy was defined according to the context of each studied region. Araripe communities have more inhabitants (about 1035 households) and a strong reliance in healers, medicinal plant specialists and midwives when compared to maroon communities. At maroon communities (about 95 households) the local health practitioners were few and, partly because of the historic repression of Afro-descendant communities and their traditions, it is possible that the recognition of local healers has been devalued over time. In Chapada do Araripe the informants were identified using the snowball method [32], starting with local leaders and ending when there were no new indications of healers, medicinal plant specialists and midwives. In the maroon communities, a census was first held among the adult residents (over 18 years old) that were willing to participate. During this stage, the residents were asked to answer a socioeconomic questionnaire, free list known plants, and indicate knowledgeable healers and people that know medicinal plants. The specialists were selected based on the indications of the informants (which included those cited by three or more people) and based on a quartile analysis, made using the program BioStat, to identify the informants that stood out based on the number of medicinal plant citations. In the Chapada do Araripe communities, data collection occurred between August 2012 and August 2013, and totaled circa 60 days of fieldwork [12]. In the maroon communities, data collection occurred between July 2013 and May 2014, and totaled circa 70 days of fieldwork [31].

In addition to questions related to socioeconomics and free listing known medicinal plants, all key informants were asked about the use of biomedicine and allopathic medicines, and about the changes on the value of healers in relation to the past (perception of the number of healers in the communities and if there is a continued demand for these specialists in the communities). The range of time considered as the past by the respondents was 30 years ago (before the 1980s), a period with intense changes in the livelihoods of the communities.

To identify the plants cited, samples were collected or photographed. The collections were processed according to the recommendations of Cunningham [33], and identified using literature [34, 35] and by consulting specialists. Specimens were deposited in the herbaria FLOR (Universidade Federal de Santa Catarina), under the numbers FLOPR53260 to FLOPR53263, and EAFM (Instituto Federal de Educação, Ciência and Tecnologia do Amazonas), under the numbers 1727 to 18041.

The interview protocol is available in S1 File. The organization of information on the use of biomedicine is available in S1 and S2 Tables and the organization on the use of medicinal plants is available in S3 and S4 Tables.

The complete information about the knowledge and use of medicinal plants in both regions has been published separately in Zank *et al*. [12, 36].

The classification of the therapeutic indications of the medicinal plants and the medications was based on the International Classification of Diseases (ICD 10) of the World Health Organization [37] and subsequently compared by percentage. We used WHO classification of diseases only for allowing some parallels between the sets of data, and not to homogenize the knowledge between regions; particularities of the local knowledge related to health practices go beyond the WHO classification and are discussed in Zank and Hanazaki [38]. The Kruskall-Wallis test was used to compare the averages of plants and allopathic medications cited by the interviewees. The form of obtaining the plants (extraction, cultivation, purchased) was compared by the category of therapeutic use (ICD 10). The Mann-Whitney test was used to compare the form of obtaining the plants, and to compare the use of medicinal plants and allopathic medicines. The quartile analysis was also used to identify the categories of use that have the highest values (above 75%) in the medicinal plants and allopathic medicines.

## Results

### Practices and traditional knowledge about health

**Fig 1:**
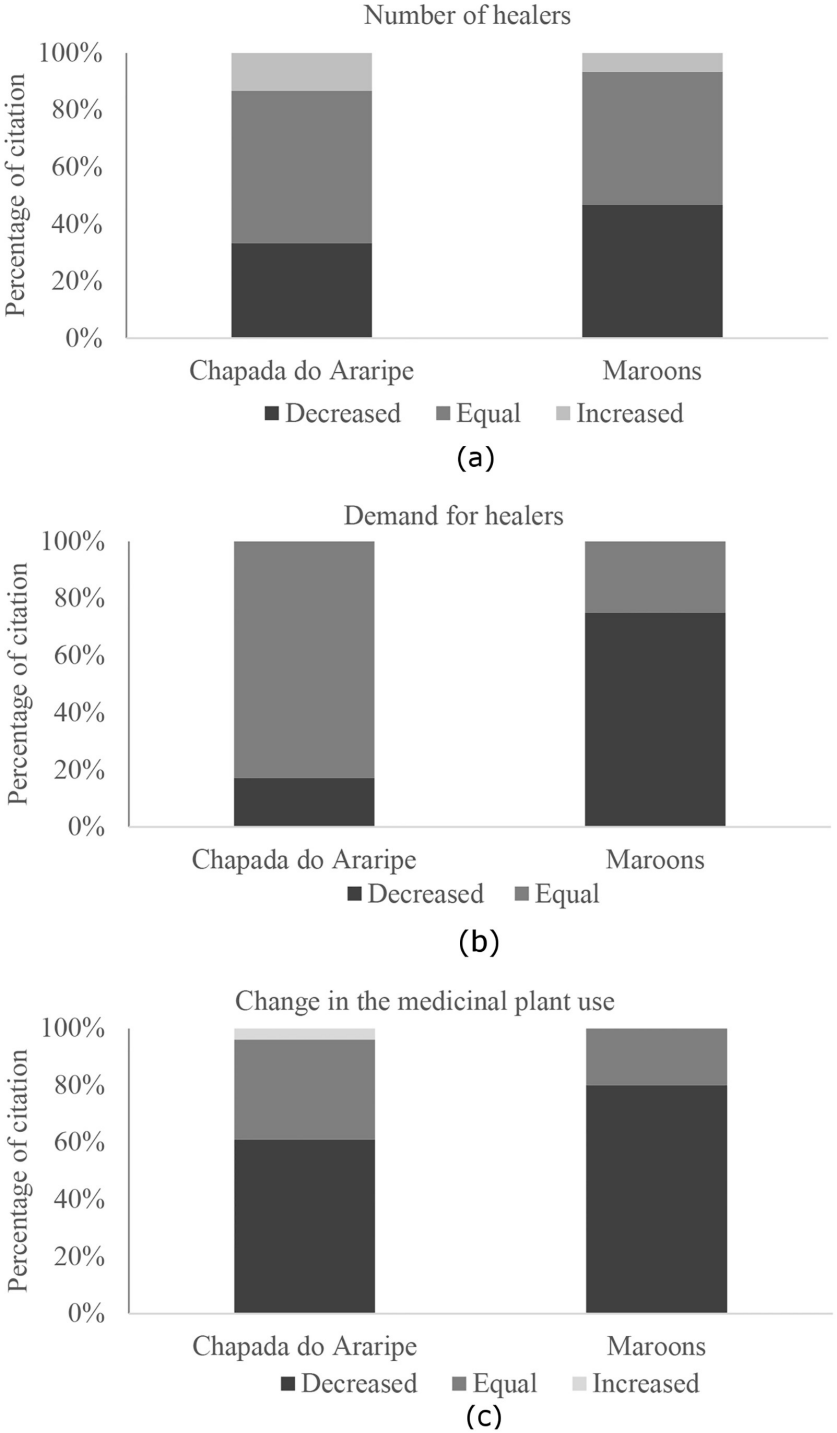
Perception of change of local health specialists from the Chapada do Araripe and maroon communities in relation to: a) number of healers (Chapada do Araripe n = 45 interviewees and maroon n = 16 interviewees), (b) demand for healers (Chapada do Araripe: n = 41 interviewees and maroon: n = 16 interviewees), and (c) medicinal plant use (Chapada do Araripe: n = 54 interviewees and maroon: n = 15 interviewees).

In the three communities of Chapada do Araripe, 66 local health specialists were identified, including 39 healers, 23 medicinal plant specialists, 1 root specialist (*raizeira*) and 5 midwives (2 of these were also healers). In the Chapada do Araripe health is still strongly related to local experts, a reflection of the maintenance of traditional livelihoods and the strengthening of local cultural identity. The experts demonstrated a broad and comprehensive understanding of human health, encompassing various influencing factors which include elements related to ancient cultural aspects (eg cultural / spiritual diseases), as well as elements related to modernity and media (eg importance of physical exercise and of avoidance of processed foods). In the Chapada do Araripe communities, 53% of the interviewees reported that the number of healers continued to be the same as in the past, and 33% reported that the number has decreased. In addition, there was a perception that the demand for healers was the same as the past (83%) (Fig 1).

Among the maroon communities, 22 local specialists were identified, including 6 healers and 16 medicinal plant specialists; 18 were indicated by the residents and the additional 4 were found after the quartile analysis. In these communities the perception of health is strongly related to the care with the body and mind, and with the access to biomedicine, which can be a reflection of the changing livelihoods. In these communities there is a high incidence of diseases, which are diagnosed solely by biomedicine (eg high blood pressure and diabetes mellitus). This incidence may be related to the rapid effects of modernization that affected such communities, and their socioeconomic situation. According to the local specialists, these health problems could be minimized through changes in eating habits and regular practice of physical exercises.

In the maroon communities there was no difference between the informants that perceived a decrease in the number of healers and those that reported no change in relation to the past (43%) (Fig 1a). The perception was that the demand for healers has decreased (75%) (Fig 1b).

The medicinal plants are principally used as home remedies and also in rituals (for blessing, sympathy and protection). In relation to the use of medicinal plants, 61% of the interviewees in Chapada do Araripe and 80% of those in the maroon communities perceived a decrease of use in relation to the past. However, for a smaller percentage of the specialists the use was the same as the past (Fig 1c). In addition, the specialists recurrently said that the younger residents did not value traditional knowledge, which created the perception that this knowledge and practices linked to health are disappearing.

There were 1390 citations of medicinal plants for the Chapada do Araripe communities, and among them 193 species were identified. For maroon communities 445 plants were mentioned and 119 species were identified.

**Fig 2:**
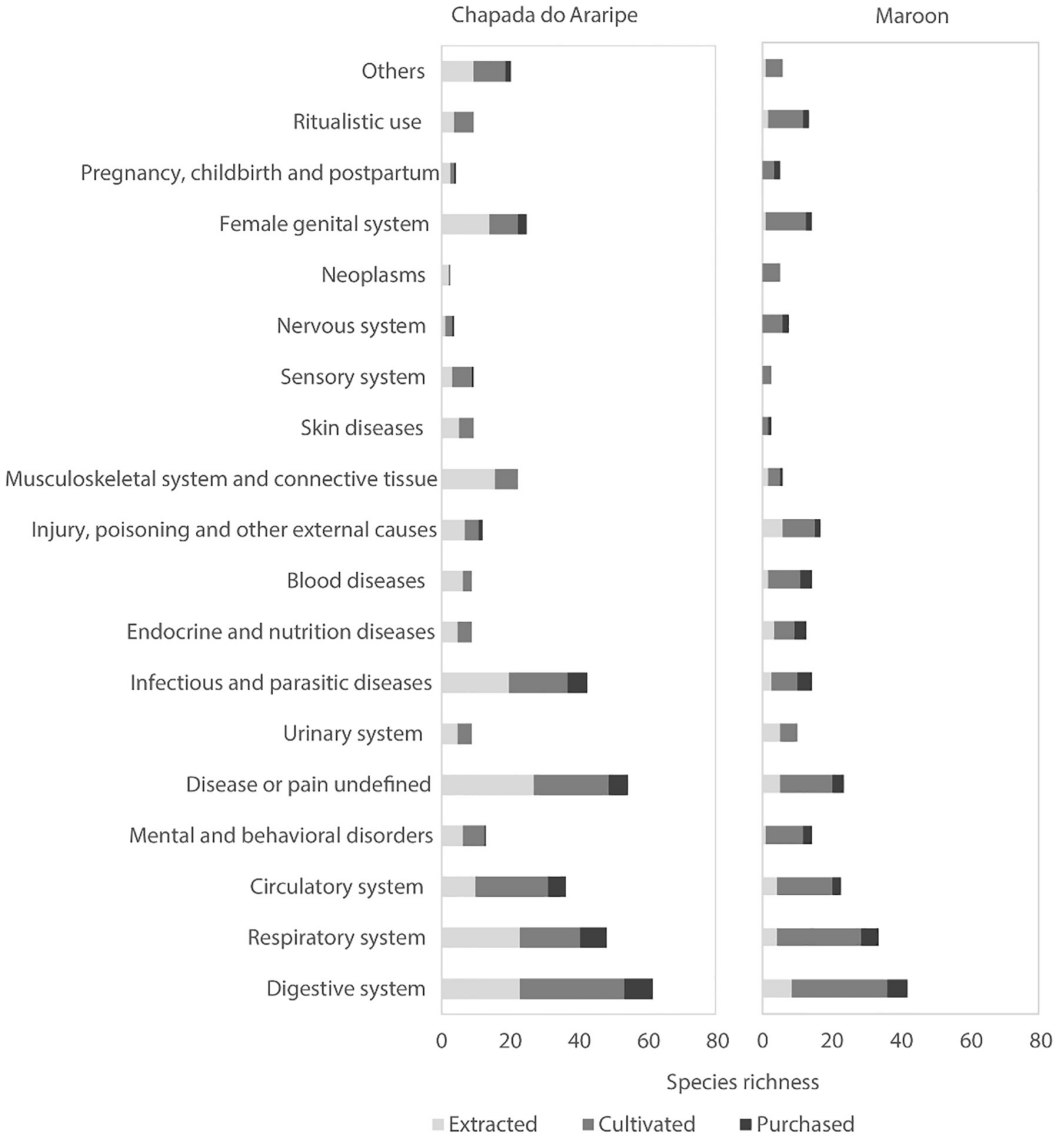
Percentage of medicinal plants cited by category of use and method of obtainment (Chapada do Araripe n = 193 plants, maroon n = 119 plants).

The plants were classified into 18 categories of therapeutic use according to the system of the body they are used to treat (Fig 2). On average, more use categories were associated with plants in the Chapada do Araripe communities (average = 4.1 and s.d. = 2.9) than among the maroon communities (average = 2.3 and s.d. = 1.8), which was significantly different (Kruskall-Wallis H = 37.03, p < 0.0001).

For Chapada do Araripe communities, a higher percentage of plants extracted from native vegetation was recorded. The number of species extracted or harvested from native vegetation was significantly different between the two regions (Mann-Whitney p <0.001) but did not differ for cultivated species(Mann-Whitney p = 0.45), or plants bought in markets (Mann-Whitney p = 0.26). In the maroon communities, cultivating plants in home gardens was the principal method of obtaining plants. Purchasing plants is important in both regions. For the maroon communities, 15 disease categories were recorded for purchased plants and in the Chapada do Araripe communities 11 categories were recorded.

The digestive, respiratory and circulatory systems, as well as undefined pains and infections, were the main categories cited for the use of medicinal plants to treat illnesses. In the Chapada do Araripe communities there was a higher citation of plants in the infectious and parasitic disease, musculoskeletal system and female genital system categories.

Among the most cited plants, 14 species were cited by more than 40% of specialists in each region, and six species are important for both regions: *Mentha* sp., *Ruta graveolens*, *Rosmarinus officinalis*, *Lippia alba*, *Cymbopogon citratus*, and *Citrus sinensis* (Table 1). These plants are mainly used to treat problems of the digestive system (nine species), and also for treatments in the categories ‘Disease or pain undefined’, ‘Infectious and parasitic diseases’, and ‘Respiratory system’, with six species cited for each. The most cited plants are cultivated (excepting for *H*. *drasticus* that is only obtained by extraction), and four of these are also extracted and purchased (Table 1).

**Table 1:** Plant species with percentage of citation higher than 40% in rural communities of the Araripe plateau and in maroon communities. CS = Circulatory system; DPU = Disease or pain undefined; DS = Digestive system; FGS = Female genital system; IPS = Infectious and parasitic diseases; MBD = Mental and behavioral disorders; NEO = Neoplasms; RS = Respiratory system; RU = Ritualistic use; SD = Skin diseases; US = Urinary system; C = Cultivated; E = Extracted; P = Purchased.

Species	Corporal System	Araripe				Maroon			
		Frequency	C	E	P	Frequency	C	E	P
*Mentha* sp.	CS; DPU; DS; IPS; RS	64%	X			59%	X		
*Ruta graveolens* L.	DPU; DS; FGS; RU	52%	X			59%	X		
*Rosmarinus officinalis* L.	CS; DPU; DS; IPS; MBD; RS; RU	48%	X			55%	X		
*Lippia alba* (Mill.) N.E.Br. ex Britton & P.Wilson	DS; MBD	41%	X			41%	X		
*Cymbopogon citratus* (DC.) Stapf	CS; DPU; DS; MBD; RS	26%	X			68%	X		
*Citrus sinensis* (L.) Osbeck	DS; MBD	33%	X		X	50%	X		X
*Plectranthus amboinicus* (Lour.) Spreng.	IPS; RS	68%	X			0			
*Himatanthus drasticus* (Mart.) Plumel	DPU; DS; NEO	41%		X		0			
*Scoparia dulcis* L.	DS; IPS; RU; SD	41%	X	X		0			
*Melissa officinalis* L.	DS; MBD; RS	0				77%	X		X
*Tanacetum parthenium* (L.) Sch.Bip.	FGS; IPS	0				50%	X		X
*Cotula australis* (Sieber ex Spreng.) Hook.f.	IPS	0				45%	X	X	
*Plantago* spp.	DPU; RS	0				45%	X	X	
*Bauhinia forficata* Link	US	0				41%	X	X	

### Coexistence between traditional practices and biomedicine

All communities have easy access to biomedicine, including health centers in or nearby the communities and health workers that attend to families. The health centers represent important means of access to biomedicine, for both regions, where people can make appointments with doctors, monitor chronic health problems and access allopathic industrialized medicines.

When questioned about the use of allopathic medicines, the majority of the interviewees (85% for Chapada do Araripe and 68% for the maroon communities) reported that they currently use this type of treatment. The maroon specialists reported using more allopathic medicines (average = 1.5 and s.d. = 1.5) than in Chapada do Araripe communities (average = 1.29 and s.d. = 1.1), but this difference was not significant (Kruskall-Wallis H = 0.0015, *p* = 0.97).

Chapada do Araripe is located in a more isolated region and lacks basic assistance (eg piped water and basic sanitation), and there the guiding role of health agents is fundamental for the reduction of some diseases, such as diarrheal diseases that impact child mortality.

**Fig 3:**
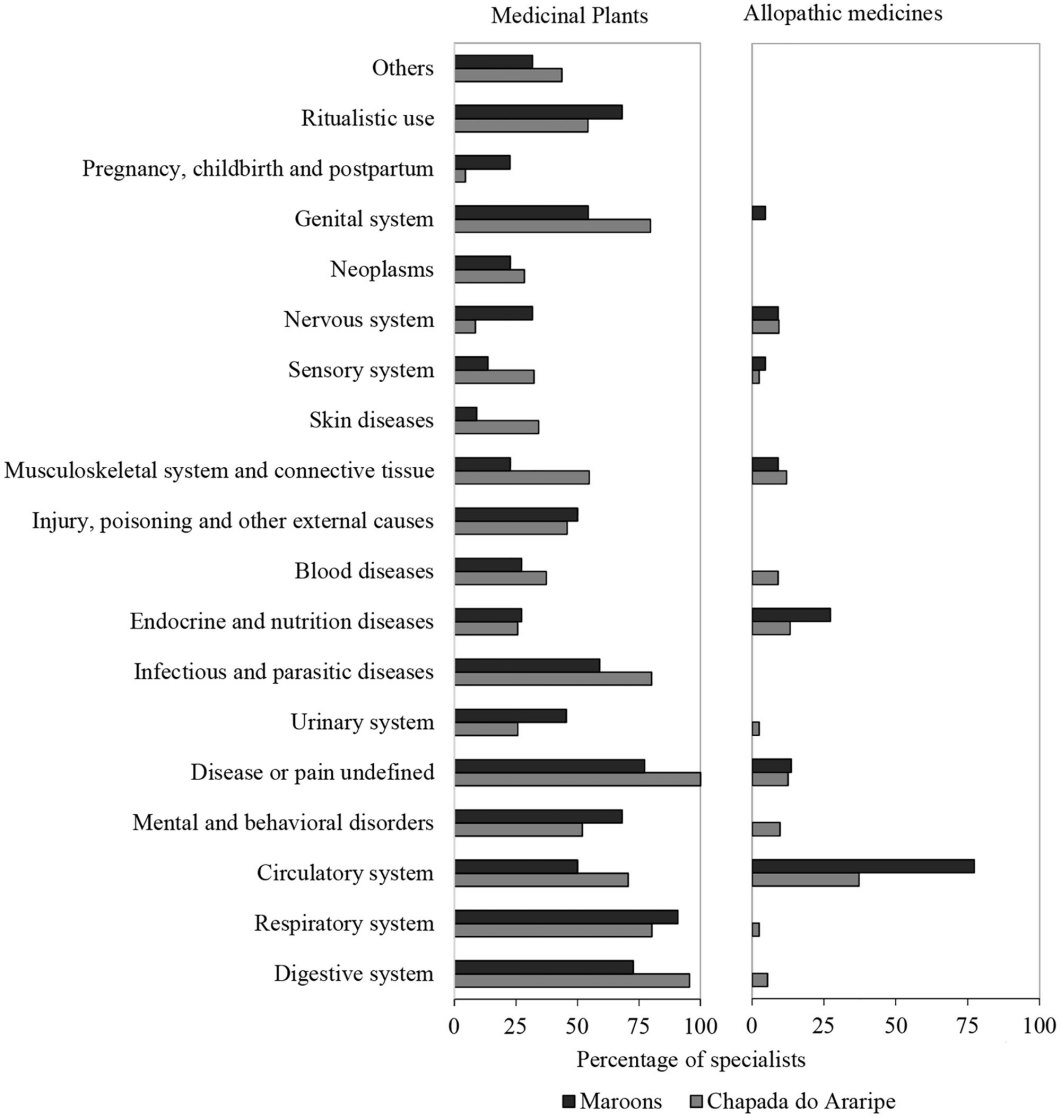
Medicinal plant knowledge and use of allopathic medicines by local health specialists in the Chapada do Araripe communities (n = 66 interviewees), in Ceará, and the maroon communities (n = 22 interviewees), in Santa Catarina.

In the maroon communities, the access to biomedicine resources is more intense due to the proximity to urban centers and the modification of traditional livelihoods. People know and use plants to treat diseases of all categories in the two regions, but allopathic industrialized medicines are used only for 11 categories of diseases in Araripe and seven categories in maroons communities (Fig 3). The difference between the two options of treatment was significant for both regions (Mann-Whitney p<0.001).

In Maroon communities, 75% of the health specialists use industrialized medicine for circulatory problems, and this number is only circa 25% for the specialists from Chapada do Araripe. Medicines used for some categories were recorded for the Chapada do Araripe communities and not for the maroon communities, such as the allopathic medicines used for the digestive system, respiratory system, mental and behavioral diseases, the urinary system and blood diseases (Fig 3). However, only in the maroon communities the use of medicines was recorded for the genital system.

The quartile analysis showed that the plants and medicines are used in a complementary way (Table 2). Medicinal plants are used in the two regions to treat health problems related to the digestive system, respiratory, and disease or pain undefined. Additionally, they are used in the Araripe region for treatment of infectious and parasitic diseases, and reproductive system, and in the maroon communities for mental and behavioral disorders, and ritualistic use. Allopathic medicines are used mainly to treat problems of the circulatory system, diseases and pains not defined, and endocrine and nutritional diseases. Furthermore, in Araripe, they are also used to treat problems of the musculoskeletal and connective system (Table 2).

**Table 2:** Categories of diseases that stand out for the use of herbal and allopathic medicines, based on the quartile analysis for the Chapada do Araripe and maroon communities. Bold numbers indicate values that are above the 75% quartile.

Categories of diseases	Medicinal plants		Allopathic industrialized medicines	
	Araripe	Maroon	Araripe	Maroon
Digestive system	**95.5**	**72.7**	5.4	0
Respiratory system	**80.3**	**90.9**	2.5	0
Circulatory system	70.6	50.0	**37.2**	**77.3**
Mental and behavioral disorders	52.1	**68.2**	9.7	0
Disease or pain undefined	**100.0**	**77.3**	**12.5**	**13.6**
Infectious and parasitic disease	**80.1**	9.1	0	0
Endocrine and nutritional disease	25.7	27.3	**13.2**	**27.3**
Musculoskeletal and connective system	54.8	22.7	**12.0**	9.0
Reproductive system	**78.7**	45.5	0	4.5
Ritualistic use	54.3	**68.1**	0	0

The category of disease or pain not defined was the only one with high values for both types of treatment (by 75% of the informants in Araripe and 100% in maroon communities). It is important to consider that there may also be simultaneous use of these two treatments. Similarly, in the circulatory system category, which had a greater citation of allopathic medicines, 28% of Araripe interviewees and 53% of maroons who reported using allopathic medicines, also cited the use of plants.

## Discussion

The two regions studied have differences in relation to environmental and cultural contexts, which also influences the way they use plant resources and industrializes medicines to treat health problems. Among these differences, we highlight a) the surrounding ecosystems: caatinga region (semi-arid) for Araripe communities, and Atlantic rainforest region for maroon communities, presenting a different set of native medicinal plants for each locality, and different climatic regimes influencing the crops and the seasonal availability of plants; b) ethnic origin of the informants: the Araripe communities are of mixed descent (European, indigenous and Afro-descendant) and Maroon communities are Afro-descendants; c) historical context: the region of the Chapada do Araripe, located in the countryside of northeastern Brazil and farther than large urban centers, remained more isolated and with fewer possibilities of development; maroon communities are located near the state capital, in a region where the mass tourism has expanded, and had a greater influence of modernization and more options in terms of economic development; and d) the regional socio-economic settings of each state are different and illustrate part of the heterogeneity among Brazilian regions: maroon communities studied here are from Santa Catarina, one of the more developed states in Brazil, while Araripe communities are located in Ceará state, in a less developed region. We also recognize that these factors are correlated and can not be isolated from each other.

On the other hand, both regions are subject to the same external processes that affected traditional health practices such as growth of the public service network. Other process that affected both regions was the improvement of the roads since the 1970s, reflecting a period of growth in Brazilian economy. In the 1990s, with the establishment of the family health programs, the action of health agents and the presence of health centers in remote areas were intensified. Thus, it is important to consider both the differences between regions, as well as the similarities in the process of coexistence between traditional medicine and biomedicine.

We observed different perceptions in relation to changes in the demand for healers and use of medicinal plants. In the maroon communities the perception of a decrease in the use of traditional practices was more expressive, whereas in the Chapada do Araripe communities no change was perceived. This difference may be a result of the maintenance of traditional livelihoods in the Araripe, whose communities have not experienced a strong modernization process when compared to the maroon communities. Perceptions about changes compared to the past are relative within each region. However, they can also indicate distinct cosmovisions of health and cure. In the case of Araripe communities, the healers, prayers, and medicinal plant specialists are widely recognized and connected through networks of exchanging knowledge and plants [38]. The same was not observed in maroon communities, and the reasons why are related to contextual differences: maroon communities are smaller, the role of healers are not as strongly recognized, and the reliance on allopathic medicine could have influenced these communities longer before the presence of health agents in the 1990s due to the proximity to urban centers.

The perception about the experts within each community was not the same. These different perceptions were also found in a study about healers from Ivaiporã, Paraná/Brazil [4]; some informants perceived a decrease in demand for healers, mainly caused by biomedicine, and others thought they were valued more and that biomedicine was complementary to traditional health practices. For many experts, biomedicine presents no threat to the practice of healers, since there are some types of diseases such as those of cultural or spiritual origin that are only treated by healers [38, 39]; and this perceptions also reflects different cosmovisions of health and cure.

In relation to knowledge of medicinal plant use, the Chapada do Araripe communities were notable for their use of extracted plants compared to the maroon communities. Medeiros *et al*. [40], in a comparison of different Brazilian ecosystems, found that in the Caatinga the use of native species is predominant where as in the Atlantic Forest the use of exotic plants is predominant. According to these authors this is related to several factors, including historical reasons because Europeans first settled in areas with Atlantic Forest and the inhabitants probably had more contact with the knowledge and plants brought by immigrants. Other factors that could have influenced this are the property system, the protection of native areas of Atlantic Forest and the resource availability hypothesis [40]. Besides that, the use of medicinal plants grown in arid environments is limited by irregular rainfall and the need to save water for other more important uses [41], thus is more interesting to use the available resources already adapted to the arid environments (eg native plants) than cultivate plants for the same purpose.

In the two regions, purchasing plants was a form of obtainment found for almost all disease categories. The medicinal plants in the Chapada do Araripe region are purchased mainly in traditional open markets [12], which are places where traditional knowledge is maintained and, on a small scale, represent the biological and cultural diversity of the region [42]. However, in the maroon communities these resources are purchased in pharmacies or supermarkets, with no direct link with the local knowledge in the production or commercialization of these plants. In general, extraction and cultivation help maintain traditional livelihoods and empower communities in the use of traditional medicines; on the other hand, purchasing medicinal plants is dependent on market availability and the financial resources of the buyer.

The medical knowledge associated with plants is focused on plants that require local cultivation and/or extraction: the six most cited plants (*Mentha* sp, *Ruta graveolens*, *Rosmarinus officinalis*, *Lippia alba*, *Cymbopogon citratus*, and *Citrus sinensis*) were shared by the two regions studied- and are exotic plants widely distributed in different Brazilian regions [35]. Bennett and Prance [43] have also observed in other Latin American countries the prevalence of common exotic species in the local pharmacopoeias.

In the region of Chapada do Araripe there is greater richness of known plants and more categories of use associated with each plant, which could indicate that the specialists in this region maintain a greater diversity of knowledge related to medicinal plants. This may be a reflection of the traditional practices that are most used in this region, in relation to the maroon communities where we observed a decrease in the demand for healers and medicinal plants. This fact can be related to the historical context of the two regions.

Despite the differences between regions, the coexistence and the complementary use of medicinal plants and allopathic medicines was observed in both. In the two regions studied, medicinal plants are used to treat problems of digestive system, respiratory system, and general pains. This has been observed in other studies in different locations and human groups, such as the Fulni-ô Indians in Northeastern Brazil [44], rural communities in the Brazilian midwest [5], and south coast [18, 20], and with indigenous Tsimane of the Bolivian Amazon [17], and in villages of Burkina Faso, West Africa [7]. Particularities of this coexistence and complementarity reflects different contexts and cosmovisions of health. For the communities of Araripe the use of plants was prominent for parasitic and reproductive system diseases (especially the "women's infections") and in maroon communities for tranquilizer and ritualistic use. These differences may be related to the incidence rates of diseases in each region, as well as to cultural aspects (eg, the use of plants for the treatment of cultural and spiritual diseases in the Afro-descendant maroon communities).

As discussed by Benitez *et al*. [45], medicinal plants are preferred to treat simpler health problems that do not require medical care, such as gastrointestinal problems and colds. In addition, medicinal plants are the principal method used for self-treatment by local communities [17, 46] and are important in primary healthcare in rural areas [16], and this is reflected in the high levels of plant use to treat primary health problems compared to the use of allopathic drugs in the studied communities.

On the other hand, in both regions the use of allopathic medicines by the experts reflects the easy access to biomedicine resources. These industrialized medicines are used principally for problems with blood pressure, general pain, and endocrine and nutritional diseases, such as diabetes mellitus. Blood pressure and diabetes mellitus are diseases that became known through biomedicine, as previously there were no means to diagnose them through traditional medicine; moreover, they are also diseases that increased their frequency in the communities as a reflection of changing livelihoods (more sedentary jobs and changes in diet). Since 2004, some industrialized medicines for high blood pressure and diabetes were included in a governmental program in Brazil that provides these medicines with low price (less than 90% of the commercial price) or at no cost for the user, upon the presentation of medical prescriptions.

The low cost of the medicinal plants was a reason given by the interviewees to use them. People also have greater autonomy to obtain medicinal plants when compared to industrialized medicines, and they are not afraid to have collateral effects as severe as allopathic medicines. On the other hand, according to the informants the allopathic medicines have a faster effect in alleviating the symptoms of disease, and for those with paid employments in the urban centers it is simpler to buy a medicine in a pharmacy, than to grow or collect plants.

Many people who mentioned the use of allopathic medicines for health problems related to the circulatory system and undefined pains also cited the use of plants. This is a phenomenon observed in other communities of Brazil [5] and of the world [47, 48] and there is a need to give attention to the possible consequences of drug interaction.

Traditional medicine plays a very important role in maintaining the health of the communities studied, being used in a complementary and simultaneous way with biomedicine. Whereas it is important that some diseases are treated by biomedicine, simpler ones (such as light diarrhea and colds) can continue to be treated with the use of plants. Therefore, the practice of traditional medicine can not be ignored by formal health systems and should be incorporated and valued to ensure the best health benefits for communities. This incorporation must consider the particularities of each location, and the existence and reliance of healers, midwives, and prayers. Giraldi *et al*. [49] reinforced the role of ethnobotany in defining public health policies to overcome the limitations of such policies regarding medicinal plants in Brazil, which can homogenize the high sociodiversity and the diverse knowledge associated with natural resources. The use of both traditional medicine and biomedicine is possible and can benefit local populations [17, 50, 51].

Even though, it is important to respect the qualifications of local health specialists that value and incorporate this knowledge into traditional safety and health practices. While these experts feel valued, they continue spreading their knowledge of traditional medicine in the communities where they operate. Traditional medicine becomes a resource for cultural affirmation in its confrontation with the dominant medical system, and its maintenance helps to maintain traditional livelihoods and conserve the local ecosystems.

## Conclusion

In this study we highlight the contrasts and similarities in two different regions of Brazil and we found that access to biomedicine does not necessarily displace traditional medicine, but that these two systems can coexist in a complementary and complex way. However, there are different situations in the coexistence of these two systems. In the Araripe region, we observed a strong maintenance of health practices, reflecting historical, cultural and environmental particularities. The distance to urban centers, the size of the communities and the networks between healers and prayers, and the availability of medicinal plants in natural environments are related to such maintenance. In the maroon communities the changes in the traditional health practices are more present, due to modernization, easy access to urban centers and urban jobs, and changes in the local livelihoods. We are assuming that in the past, maroon and Araripe communities depended more in their self reliance for health care, but one of the limitations of this study is that we do not have the precise scenario of the local health practices before the 1970s. In any case, there is a tendency for the two regions of a decrease in the number of healers, demand for healers, and use of medicinal plants, especially among maroons.

In addition to the differences in the two regions, knowledge about medicinal plants and the use of medicines was complementary in the two regions, where medicinal plants are principally used to treat simpler diseases, such as digestive and respiratory disease and general pains, and allopathic medicines are principally used for problems with blood pressure, general pain and endocrine and nutritional diseases.

Cultivation and harvesting are still the main way of obtaining plants in both regions, and this favors the maintenance of traditional knowledge. Changes in local livelihoods may change the availability and access to plant resources, when people skip from rural activities to urban ones. However, a local cosmovision abouth health in which prayers, healers and medicinal plant specialists are valued can counterbalance these changes, and may vary depending on the socioeconomic context. This study also found that there was knowledge and appreciation for traditional health practices in both regions. It is important to create strategies to train health professionals to work under these complementary conditions of therapy and to respect the different forms of knowledge in order to seek a higher quality of the healthcare for local communities.

## Supporting information

S1 File English interview protocol. Interview protocol used in the study.
(PDF)

S1 Table Part of Araripe allophatic medicine. Organization of information on the use of allophatic medicine of Araripe informants.
(XLS)

S2 Table Part of maroon allophatic medicine. Organization of information on the use of allophatic medicine of Maroon informants.
(XLS)

S3 Table Part of Araripe free list. Organization of information on the use and the way of obtaining medicinal plants of Araripe informants.
(XLS)

S4 Table Part of maroon free list. Organization of information on the use and the way of obtaining medicinal plants of Maroon informants.
(XLS)
