# Supplementary material for: The coexistence of traditional medicine and biomedicine: A study with local health experts in two Brazilian regions
Source: PLoS One. 2017 Apr 17;12(4):e0174731. doi: 10.1371/journal.pone.0174731 (PMC5393556; doi:10.1371/journal.pone.0174731)
Supplement: S1 File — Interview protocol used in the study. (PDF) [file pone.0174731.s001.pdf]

INTERVIEW PROTOCOL - LOCAL SPECIALISTS

Researcher: Sofia Zank

Name of the interviewer: ________________________________Date: ________________

Area of Study: ________________________ Community:_________________________

House number:______________________Number of the interview_________________

1. Name:______________________________*(information kept anonymously. The name is just for the interviewer´s reference)* 2. Gender: ________ 3. Age:__________

4. Occupation/activity_________5. How long have you lived here?______________

6. Expert in:____

7. What local health practices take place in the community (healers / raizeiros / rituals)? Do you perceive changes (number of specialists, demand, valorization ...) in these practices over time?

8. Has the use of medical resources for the health processes changed? Did any changes occurred in access to medical resources?

9. Do you take industrialized medicines? Which (type of disease)?

☐ No ☐ Yes

Health problem:____________________

Health problem:____________________

Health problem:____________________

10. Indication of other experts:

11. What plants do you know?

N°	Environment / landscape	Medical resource<br><br>Local name	Use	Used part	Method of preparation	Obtaining Form	Collection
